# Explaining mobilization for revolts by private interests and kinship relations. A response to the comment by Nieva

**DOI:** 10.1177/10434631251324075

**Published:** 2025-03-13

**Authors:** Niccolò Giorgio Armandola, Malte Doehne, Katja Rost

**Affiliations:** 27217 University of Zurich, Switzerland

**Keywords:** Private interests, revolts, kinship relations, social networks, civil uprisings

## Abstract

We appreciate Ricardo Nieva’s thoughtful engagement with our paper and his proposal to interpret our case study through the lens of his theoretical framework. Indeed, we noted the potential of such a connection in the original paper, and thank Professor Nieva for taking up our suggestion. This response aims to identify points of overlap between our accounts, clarify key conceptual distinctions, and explore the implications of each approach for understanding the dynamics of mobilization.

## Introduction

We appreciate Ricardo Nieva’s (2024) thoughtful engagement with our paper and his proposal to interpret our case study through the lens of his theoretical framework (Nieva, 2019, 2021). Indeed, we noted the potential of such a connection in the original paper (Armandola et al., 2024, Footnote 2), and thank Professor Nieva for taking up our suggestion. This response aims to identify points of overlap between our accounts, clarify key conceptual distinctions, and explore the implications of each approach for understanding the dynamics of mobilization.

Our empirical study investigates the dynamics of mobilization for revolts using the case of Basel in 1691, when a well-documented local uprising took place, referred to as the *Einundneunziger Wesen* (Schweizer, 1931). Following tensions with neighboring France, insurgent citizens besieged the city hall on March 24th, 1691, and ultimately took control of the city’s parliament (Schweizer, 1931). Through an analysis of comprehensive genealogical data on Basel’s citizenry, we find that distant kin of the elite were more likely to join the revolt than close kin of the rebelling faction. Our account thus underscores the critical role of kinship relations as a driver of collective action.

Nieva (2024) proposes an alternative interpretation centered on coalition formation and the pivotal role of an ‘enforcer’ elite that controls the distribution of output (e.g., by defining and enforcing property rights). According to Nieva’s account, society consists of (nonproductive) enforcers and peasants, the latter generating the output that is subject to distribution. Individuals form coalitions in their efforts to gain control over the distribution of outputs. Marginal labor productivity emerges as a key determinant of the composition and size of the ruling coalition, comprising enforcers and peasants, in contrast to a marginalized and exploited faction of outsiders. Specifically, Nievas model suggests a stable equilibrium in which even weak enforcers may become part of the ruling elite, provided they are too weak to challenge the dominant enforcers, if overall marginal productivity remains positive. However, shifting circumstances may incentivize weak enforcers to enter into coalitions that upend the status quo, creating conditions ripe for uprisings and revolts. Consequently, Nieva’s account emphasizes power differentials, coalition formation, and institutional roles in shaping mobilization dynamics. Drawing on this account, Nieva explains the *1691 Wesen* as a consequence of changes in the power dynamics between the leading elite and the marginal elite at the time (Nieva, 2024).

A central point of convergence between our framework and Nieva’s lies in their theoretical foundation. Both are rooted in Tullock’s private interest theory (1971), which underscores the role of individual incentives in driving mobilizing for revolts. Building on this theory, both approaches employ formal models to predict whether individuals will participate in an attempt to overthrow the ruling elite (Armandola et al., 2024; Nieva, 2024). However, a crucial divergence arises in how we use of the respective models. In our case, we use the model to generate hypotheses that we then test against a historical dataset, whereas in Nieva’s account, we propose that the model essentially serves as a behavioral microfoundation for explaining social outcomes. This invites a deeper comparison of the two approaches along three dimensions: (1) Nieva’s categorical classification of agents versus our continuous relational perspective; (2) the methodological challenges both approaches face in connecting to historical data; and (3) the focus of either account on macro-, meso-, and micro-levels of explanation, respectively. While these differences may appear subtle, they underpin key epistemic divergences in how either framework interprets the social drivers of mobilization to revolts.

First, Nieva’s application to the historical case study builds on the observation that a subgame perfect equilibrium is possible in which the weaker of two enforcers is included in the ruling elite but gets a lower payoff than the strong enforcer (Nieva, 2024). It is this agent who, if given a chance to improve their situation, will mobilize to challenge the status quo if and when circumstances change to their advantage. Nieva’s theory thereby takes the individual’s role, that is, their status as strong or weak enforcers (or as peasant) as predictive of their ability and willingness to challenge the status quo. Agency is thereby tied closely to role assignments and to how the individuals of any one faction fare in the shifting landscape of power. In contrast, the relational account that we develop in our paper considers the relative likelihood that an individual will affiliate with either faction (elite or rebels) based on their social proximity to either group, as operationalized by kinship. This metric establishes a graded distinction, treating mobilization less as an individual’s calculated choice and more as a product of kinship and circumstance. This basic distinction between a categorical, role-based account of agency and a relational/graded account of social proximity has implications for empirical application and subsequent interpretation. In the spirit of rendering these distinctions productive, we will juxtapose both approaches with the intent of (a) highlighting the benefits and drawbacks of either approach in application to concrete empirical settings, and (b) emphasizing the value of Nieva’s framework as providing a macro-level context and of our framework in framing a meso- and micro-level analyses. Taken together, these three levels – macro, meso, *and* micro – suggest a basis for an integrated explanation for the mobilization of revolts.

Second, subjecting Nieva’s model to systematic empirical analysis requires assigning role-membership to the actual individuals who participated in the uprising, notably in distinguishing members of the strong and weak elite – an essential yet complex task. As it turns out, clarifying the distinction between strong and weak elite is challenging in the historical empirical context, making Nieva’s account challenging to apply at the fine-grained level of considering actual individuals and their specific situations. Surely some individuals stand out as plausible representatives of either group. For instance, Nieva suggests that Jacob Henric-Petri be considered a member of the weak faction within the elite as he initially held a marginal parliamentary position (Nieva, 2024: p. 4), whereas affirmed politicians in elevated office, such as Christof Burckhardt, might represent the strong faction within elite. Nieva suggests that this established an equilibrium in which Henric-Petri, despite being nominally part of the elite, had a diminished payoff, incentivizing him to mobilize and challenge the status quo when the Swiss peasant war of 1653 weakened the dominant elite (Nieva, 2024: p. 5f.). While this classification may hold in Henric-Petri’s case, single observations lack the quantitative depth needed to support claims about the general mobilization tendencies of larger groups. However, gathering systematic data on key indicators like elite status, for example, via occupations or socioeconomic endowment, is challenging across large populations, even in a case that is as well-researched as early modern Basel (Geweke et al., 2022). Our kinship-based approach offers a scalable solution, leveraging relational data to capture broader patterns and insights into elite proximity through network analysis. Unlike approaches that rely on isolated cases or predefined categories, this method evaluates the elite status of many individuals relative to one another. By operationalizing social distance in terms of individuals’ positions in kinship networks, we move beyond the limitations of predefined roles, allowing for robust and scalable analyses of power dynamics and mobilization patterns.

The third point of our response takes on a conciliatory note by acknowledging the complementarity of either approach. We propose that Nieva’s account is particularly well-suited to frame macro-level explanations predicting whether or not a marginalized elite, if it exists, will grasp for power – and when it will do so. For the specific case of Basel in 1691, Nieva identifies a critical point when “the economic position of the ruling elite […] became weaker relative to the marginalized elite and other citizens in the historical period before the uprising” (Nieva, 2024: p. 2). To substantiate his reading, Nieva marshalls “historical evidence showing that the marginalized elite and other citizens became relatively more powerful and excluded the oligarchy in the winning coalitions”. We appreciate the macro-level evidence that Nieva has presented in support of his account: the destabilizing effects of the Swiss peasant war of 1653 on the established oligarchic elite, rapid population growth from 1500 to 1700, and the 30 Year’s War from 1618 to 1648. Our own reading of the situation supports that these macro-level shocks did indeed create a local environment that was conducive to challenges of the status quo. This amounts to explaining why an uprising took place where and when it did, that is, in Basel in 1691. Thus, Nieva’s model lends itself to a reconstruction of the broader historical, material, and social context that led to the uprising. Doing so brings into focus the broader historical context that prompted individuals to affiliate with the conflicting parties. This is a worthy endeavor, but it differs from the more specific aim of our paper, which seeks of uncover the (marginal) effect of kinship relations on mobilization.

The explanatory goal of our paper is not to predict why and whether the uprising would occur but to model, predict, and explain individuals’ affiliation choices as that uprising unfolded, given individuals’ relative positions in the network of kinship relations. Put differently, whereas Nieva’s account identifies the macro-level circumstances that trigger the social dynamic in question, ours is directed towards uncovering the faultlines along which that dynamic plays out at the meso-level of individuals who are enmeshed in social- and kinship relations. This transition from macro- to meso-level (or from micro- to meso-level, for that matter!) requires systematic consideration and modeling of relational dynamics shaping social processes (see Doehne et al., 2024a, 2024b for an attempt to theorize how macro-environments shape network dynamics and vice versa). Indeed, the uprising was in response to multiple perceived problems at the same time. In the years leading up to the revolt, the city had become deeply embroiled in familial and socioeconomic webs that crystallized an emergent oligarchy (Doehne et al., 2023). These dynamics extended well beyond the economic and political sphere to include consolidation of power in the assignment of religious offices and in the university (Rost and Doehne, 2020), shifts in marriage strategies (Armandola et al., no date), the evolution of family clans (Armandola, 2024), and even mobility on the local housing market (Geweke & Armandola, no date). Thus, the *1691 Wesen* was not simply the product of a shifting power dynamic, but consequence of a crisis of legitimacy, of social opportunity, and of institutional representation. These complexities underscore the need to look beyond economic conditions alone, highlighting the relational dynamics of kinship and elite reproduction that shaped the broader socio-political landscape. Elite reproduction is not simply a matter of coalition-building or power balancing, as a purely game-theoretical model might imply. Rather, it reflects the embeddedness of elite families within complex webs of affiliations.

Against this backdrop, our paper’s analytical design and focus on the marginal effect of kinship distance has two primary advantages. First, it demonstrates that kinship effects remain significant net of alternative explanations. Second, elite reproduction is a multifaceted process that also relies on strategic marriages. Our study lays groundwork for studying how kinship ties often act as conduits for economic strategies, including alliances formed through marriage (Armandola et al., no date; Padgett and McLean, 2006). Our findings reveal that kinship ties provide a framework through which the pursuit of private interests is mediated and tempered. For instance, distant kin of the elite can leverage familial connections to maximize private rewards while avoiding social exclusion. By examining kinship as a proxy for social ties, our analysis offers a targeted explanation of how private interests are channeled through well-established social networks. Investigating ensuant dynamics from a relational perspective generates valuable insights into how different types of social networks amplify the mobilization potential of marginalized elites. We hope our response underscores the value of a relational perspective—one that situates individual agents within their broader networked contexts—in enriching the study of mobilization.
